# Biomarkers for prognostic functional recovery poststroke: A narrative review

**DOI:** 10.3389/fcell.2022.1062807

**Published:** 2023-01-09

**Authors:** Jack Jiaqi Zhang, Dalinda Isabel Sánchez Vidaña, Jackie Ngai-Man Chan, Edward S. K. Hui, Kui Kai Lau, Xin Wang, Benson W. M. Lau, Kenneth N. K. Fong

**Affiliations:** ^1^ Department of Rehabilitation Sciences, The Hong Kong Polytechnic University, Kowloon, Hong Kong SAR, China; ^2^ Department of Imaging and Interventional Radiology, The Chinese University of Hong Kong, Shatin, Hong Kong SAR, China; ^3^ Department of Psychiatry, The Chinese University of Hong Kong, Shatin, Hong Kong SAR, China; ^4^ Division of Neurology, Department of Medicine, School of Clinical Medicine, LKS Faculty of Medicine, The University of Hong Kong, Pokfulam, Hong Kong SAR, China; ^5^ State Key Laboratory of Brain and Cognitive Sciences, The University of Hong Kong, Pokfulam, Hong Kong SAR, China; ^6^ Department of Rehabilitation Medicine, Clinical Medical College, Yangzhou University, Yangzhou, China

**Keywords:** stroke, functional recovery, molecular biomarkers, blood biomarkers, neurophysiology, neuroimaging, multimodal biomarkers

## Abstract

**Background and objective:** Prediction of poststroke recovery can be expressed by prognostic biomarkers that are related to the pathophysiology of stroke at the cellular and molecular level as well as to the brain structural and functional reserve after stroke at the systems neuroscience level. This study aimed to review potential biomarkers that can predict poststroke functional recovery.

**Methods:** A narrative review was conducted to qualitatively summarize the current evidence on biomarkers used to predict poststroke functional recovery.

**Results:** Neurophysiological measurements and neuroimaging of the brain and a wide diversity of molecules had been used as prognostic biomarkers to predict stroke recovery. Neurophysiological studies using resting-state electroencephalography (EEG) revealed an interhemispheric asymmetry, driven by an increase in low-frequency oscillation and a decrease in high-frequency oscillation in the ipsilesional hemisphere relative to the contralesional side, which was indicative of individual recovery potential. The magnitude of somatosensory evoked potentials and event-related desynchronization elicited by movement in task-related EEG was positively associated with the quantity of recovery. Besides, transcranial magnetic stimulation (TMS) studies revealed the potential values of using motor-evoked potentials (MEP) and TMS-evoked EEG potentials from the ipsilesional motor cortex as prognostic biomarkers. Brain structures measured using magnetic resonance imaging (MRI) have been implicated in stroke outcome prediction. Specifically, the damage to the corticospinal tract (CST) and anatomical motor connections disrupted by stroke lesion predicted motor recovery. In addition, a wide variety of molecular, genetic, and epigenetic biomarkers, including hemostasis, inflammation, tissue remodeling, apoptosis, oxidative stress, infection, metabolism, brain-derived, neuroendocrine, and cardiac biomarkers, etc., were associated with poor functional outcomes after stroke. However, challenges such as mixed evidence and analytical concerns such as specificity and sensitivity have to be addressed before including molecular biomarkers in routine clinical practice.

**Conclusion:** Potential biomarkers with prognostic values for the prediction of functional recovery after stroke have been identified; however, a multimodal approach of biomarkers for prognostic prediction has rarely been studied in the literature. Future studies may incorporate a combination of multiple biomarkers from big data and develop algorithms using data mining methods to predict the recovery potential of patients after stroke in a more precise way.

## Introduction

Functional recovery following stroke is a complex process involving various biological events and systematic reorganization of brain structure and functions. Previously, most studies about poststroke functional recovery focused on the motor domain, especially for the paretic upper extremity. Most stroke patients could restore nearly 70% of their lost upper extremity motor impairment in the first three to 6 months after stroke, which is known as the proportional recovery rule (Prabhakaran et al., 2008). The proportional recovery rule implies that patients with stroke experience some degree of predictable functional regains due to spontaneous neurological recovery, and the level of initial motor impairment explains a large proportion of variance in the amount of recovery (Krakauer and Marshall, 2015). This rule has also been observed in other recovery domains of stroke-related deficits, such as lower extremity motor ability (Smith et al., 2017), visuospatial neglect and aphasia (Marchi et al., 2017). However, some researchers point out that the proportional recovery rule is mainly driven by mathematical coupling, and therefore, cannot be used for precise prediction of functional prognosis after stroke (Hawe et al., 2018).

Precise prediction of functional recovery following stroke may benefit from using prognostic biomarkers. Typically, prognostic biomarkers are measured at various stages after stroke onset, i.e., the acute/early subacute stage of stroke – less than 4 weeks after stroke onset, and late subacute/early chronic stage of stroke – three to 6 months after stroke (Boyd et al., 2017). Biomarkers can be derived from systems neuroscience approaches, such as neuroimaging and neurophysiological measures of the brain’s structural and functional state. On the other hand, molecular biomarkers are also becoming a complementary assisting tool for predicting poststroke prognosis (Park et al., 2013). Molecular biomarkers can quantify the level of damage to neurons and glial cells, thereby providing insight to the mechanisms underlying injury (Maas and Furie, 2009; Dharmasaroja et al., 2012). Therefore, biomarkers collected from systems neuroscience and molecular/cellular neuroscience approaches provide comprehensive structural and functional profiles of neurons/neural systems, potentially useful for the prediction of poststroke functional prognostication.

Functional outcomes after stroke can be rated using a variety of clinical measures. According to the International Classification of Functioning, Disability, and Health, outcome measures can be categorized into three domains: body function and structure, activities, and participation (World Health Organization, 2001). The National Institutes of Health Stroke Scale (NIHSS) is a quantitative measure of stroke-related neurologic deficits, which is considered the gold standard for acute stroke (Brott et al., 1989). The Fugl-Meyer assessment (FMA) is a stroke-specific assessment for motor impairment (Fugl-Meyer et al., 1975). These impairment-based indexes focus on the alteration of body function and structure after stroke. The Barthel index (BI; Collin et al., 1988) and the modified Rankin scale (mRS; Bamford et al., 1988) are frequently used for assessing the level of dependence caused by neurological disability. In addition, some assessments are activity-oriented, for example, the action research arm test (ARAT; Lyle, 1981) is often used to assess the capacity to perform functional activities with the paretic upper extremity.

**FIGURE 1:**
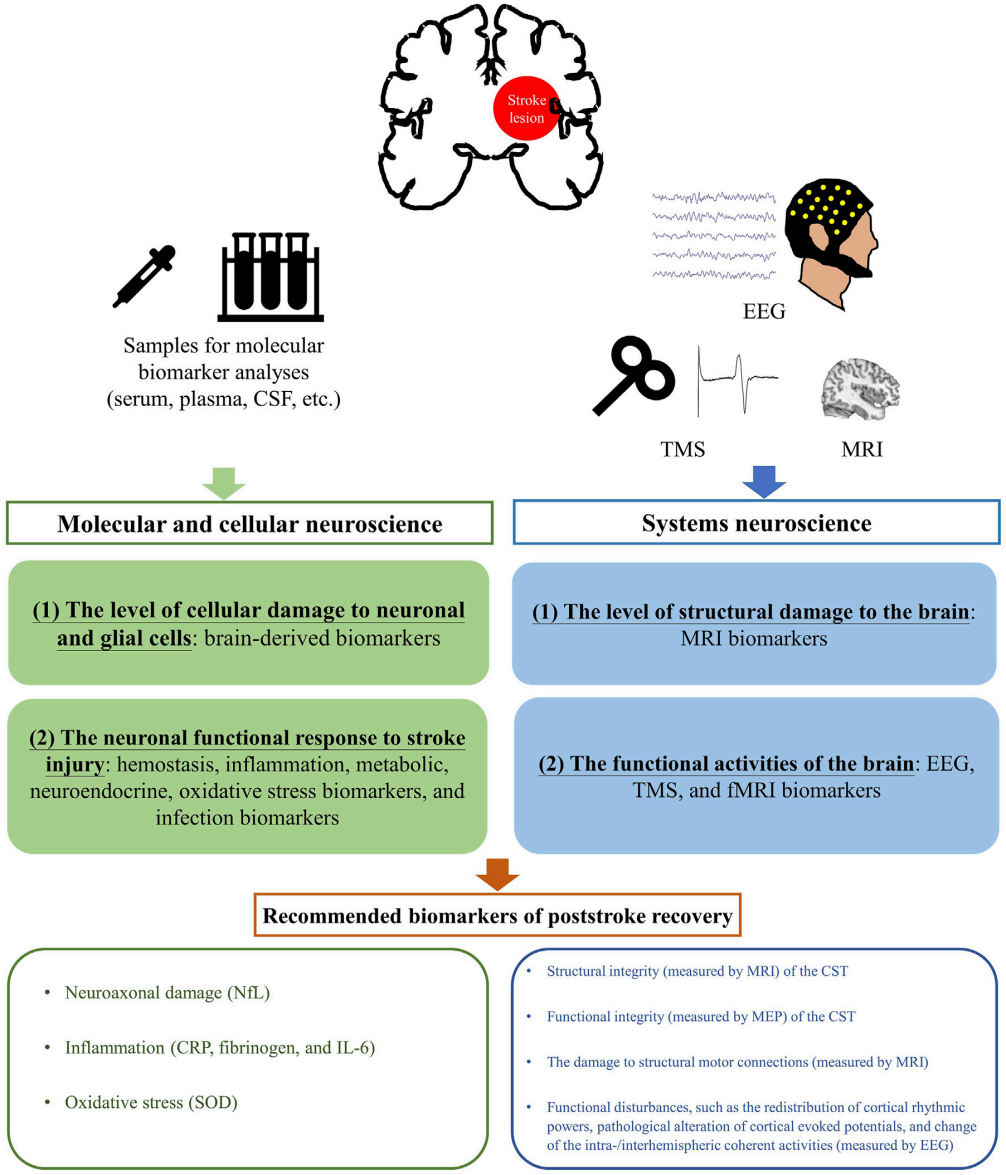
A schematic diagram of an integrative framework on the recommended biomarkers of poststroke recovery based on the review. Abbreviations: CRP, C-reactive protein; CSF, Corticospinal fluid; EEG, Electroencephalography; fMRI, Functional magnetic resonance imaging; IL-6, Interleukin-6; MEP, Motor evoked potential; MRI, Magnetic Resonance Imaging; NfL, Neurofilament light chain; SOD, Superoxide dismutase; TMS, Transcranial magnetic stimulation.

A focused review on prognostic biomarkers of stroke recovery collected using different investigative approaches has not been conducted before. Therefore, the objective of this narrative review was to review and discuss potential biomarkers to predict poststroke functional recovery using both molecular neuroscience (molecular, genetic, and epigenetic biomarkers) and systems neuroscience approaches (neurophysiological and neuroimaging biomarkers) under an integrative framework (Figure 1).

## Methodological considerations of this review

First, biomarkers included in this review fulfilled one of the two following criteria: the biomarker was: 1) independently associated with the outcome, and 2) could be used to classify patients with good or poor prognostic outcomes. Second, this review focused on prognostic biomarkers that can predict the amount of spontaneous neurological recovery after acute stroke, i.e., within the first 14–30 days to the first three to 6 months after stroke, rather than being used as a diagnostic (related to the presence of stroke) or prescriptive tool (indicative of the amount of training provided to patients). Last but not least, in order to keep the review more focused, we focused on prognostic biomarkers that were predictive of functional outcomes based on the evaluation of overall neurological deficits (NIHSS), physical disability, and functional independence (mRS and BI), or motor domain (FMA and ARAT). Biomarkers associated with cognition and language-specific outcomes, as well as clinical endpoints such as mortality and reoccurrence of stroke have been excluded. The current review is a narrative review; thus, a non-systematic approach was used to scan relevant studies. Briefly, relevant literature was collected through searches using databases and search engines such as PubMed, Scopus, and Google Scholar to retrieve studies related to biomarkers for stroke prognosis and functional recovery using the following keywords: “stroke” “recovery”, “stroke prognosis”, “functional recovery”, “stroke biomarkers”, “electroencephalography”, “transcranial magnetic stimulation”, “magnetic resonance imaging”, “blood biomarkers”, “molecular biomarkers”.

## Biomarkers collected using systems neuroscience approaches

Studies using systems neuroscience approaches have demonstrated several plausible poststroke recovery mechanisms, the more distinct theoretical framework is the interhemispheric imbalance or hemispheric rivalry model. According to the model, a unihemispheric stroke lesion suppresses the activity of the ipsilesional hemisphere, resulting in a reduction of inhibition from the ipsilesional hemisphere to the contralesional hemisphere. The disinhibition of the contralesional hemisphere then causes a relative increase in its activity and a stronger inhibition from the contralesional hemisphere to the ipsilesional hemisphere, eventually leading to interhemispheric imbalance (Murase et al., 2004). A greater interhemispheric imbalance initially after stroke may potentially predict poor prognosis (Murase et al., 2004; Saes et al., 2021). Another poststroke recovery model - the vicariation model, suggests that the activity of the contralesional hemisphere may allow compensatory/adaptive neuroplasticity, potentially to support post-stroke functional recovery (Bütefisch et al., 2005). Both models highlight the importance of interhemispheric activities in modulating poststroke recovery. Thus, structural and functional measures of both hemispheres obtained from neurophysiological and neuroimaging tools have been extensively used to provide relevant biomarkers for poststroke prognostic prediction. A list of neurophysiological and neuroimaging biomarkers for poststroke prognostic prediction is presented in Table 1.

**TABLE 1:** Neurophysiological and neuroimaging biomarkers for stroke prognosis.

Type of biomarker	Biomarker	Measurement time points (biomarker)	Analytical method	Type of stroke	Functional outcomes	Measurement time points (functional outcome)	Main findings	References
Neurophysiological biomarkers (EEG/MEG)	BSI-delta	Baseline (<3 weeks), Week 5, Week 12, Week 26	Frequency domain analysis	Ischemic stroke	NIHSS, FMA-UE	Baseline (<3 weeks), Week 5, Week 12, Week 26	(1) Directional BSI-delta was negatively correlated with FMA-UE, which was borderline significant after correction for time.	Saes et al. (2020)
							(2) Directional BSI-delta was positively correlated with NIHSS, and the association became stronger after correction for time.	
	BSI-theta	Baseline (<3 weeks), Week 26	Frequency domain analysis	Ischemic stroke	FMA-UE	Baseline (<3 weeks), Week 26	BSI-theta was the strongest predictor of FMA-UE at week 26, with higher BSI-theta value reflecting more impairment 6 months after stroke.	Saes et al. (2021)
	delta/alpha ratio	Baseline (<3 weeks), Week 5, Week 12, Week 26	Frequency domain analysis	Ischemic stroke	NIHSS, FMA-UE	Baseline (<3 weeks), Week 5, Week 12, Week 26	Delta/alpha ratio over the affected hemisphere showed a trend toward a negative association with FMA-UE scores.	Saes et al. (2020)
	SSEP	Baseline (<1 week)	Time-domain analysis	Ischemic and hemorrhagic, supratentorial stroke	mRS	Baseline, 6 months	Pathological alterations of the SSEP (absence of cortical N20 response, abnormal bilateral N20-P25 amplitude ratio, Cant grading 3, Judson grading 3, or Haupt grading 3 to 4) were predictive of unfavorable outcomes 6 months after stroke.	Su et al. (2010)
	Movement-related ERD	Baseline (7-14 days after stroke onset), 1 month, 2 months, 4 months	Time frequency domain analysis	Ischemic stroke	NIHSS, FMA-UE	Baseline (7–14 days after stroke onset), 1 month, 2 months, 4 months	(1) Recovered patients showed a significant increase of ipsilesional ERD and/or a decreasing trend of contralesional ERD.	Tangwiriyasakul et al. (2014)
							(2) The only non-recovery patient showed an increase in ERD over the contralesional hemisphere and ERD over the ipsilesional hemisphere remained absent.	
	Movement-related beta ERD/Postmovement beta ERS	Baseline (2–4 weeks), Week 5, Week 12	Time frequency domain analysis	Ischemic stroke	FMA-UE, ARAT	Baseline (2–4 weeks), Week 5, Week 12	(1) Ipsilesional M1 beta ERD was significantly positively correlated with concurrent FMA-UE scores at all three time points.	Tang et al. (2020)
							(2) Ipsilesional beta ERS at baseline was positively correlated with FMA-UE scores at Week 12.	
	FC-delta (M1-M1)	Admission (5–71 days), Discharge (10–116), Day 90	Coherence analysis	Ischemic and hemorrhagic stroke	FMA-UE, FIM motor scores	Admission (5–71 days), Discharge (10–116), Day 90	A decrease in M1-M1 coherence at delta band occurring correlated with FIM-motor scores and FMA-UE improvement during the inpatients’ stay.	Cassidy et al. (2020)
Neurophysiological biomarkers (TMS)	MEP	Baseline (<1 week), 6 months	Time domain analysis	Ischemic stroke	MRC, BI	Baseline (<1 week), 6 months	The patients with present MEP evoked by the ipsilesional M1 stimulation showed better motor and functional recovery than those without.	Escudero et al. (1998)
	CCT	Baseline (<1 week), 6 months	Time domain analysis	Ischemic stroke	MRC, BI	Baseline (<1 week), 6 months	The degree of recovery was better in patients with normal CCT than in those with delayed CCT.	Escudero et al. (1998)
	SICI	Day 5–8, Day 30, Day 90	Time domain analysis	Ischemic and hemorrhagic stroke	NIHSS, Lindmark scale, BI	Day 5–8, Day 30, Day 90	At 3 months post stroke, patients with lower BI scores at baseline (BI < 60) still showed a reduction of SICI in the unaffected M1 compared to the healthy controls, whereas the SICI in the unaffected M1 was increased to normal values in patients with higher BI scores (BI ≥ 60) at baseline.	Manganotti et al. (2008)
	IHI (during the premovement phase)	Week 1, Week 4, Week 12, Week 24, Week 52	Time domain analysis	Ischemic stroke	FMA-UE, Strength of finger flexion, Finger individuation index	Week 1, Week 4, Week 12, Week 24, Week 52	The premovement IHI was normal in stroke patients during the acute and subacute period, and became abnormal (i.e., the contralesional M1 remained inhibitory to the ipsilesional M1 prior to the movement onset) in patients at the chronic stage. The longitudinal emergence of abnormal premovement IHI was negatively correlated with the improvement of hand functions in the finger individuation task.	Xu et al. (2019)
Neurophysiological biomarkers (TMS-EEG)	TMS-evoked potential	Baseline (<14 days)	Time domain analysis (LMPF, the number of deflections), time-frequency domain analysis (the natural frequency to TMS)	Ischemic stroke	Composite recovery score (MI, ARAT, and grip force), NIHSS	Baseline (≤14 days), 3–6 months	(1) A positive correlation between the numbers of deflections of the TMS-EEG response and better motor recovery.	Tscherpel et al. (2020)
							(2) The general neurological recovery (changes of the NIHSS scores) was significantly linked to all TMS-EEG outcomes.	
Neuroimaging biomarkers (MRI)	Lesion (PLIC, CS, and CR)	Baseline (<12 h), Day 3, Day 30	DTT	Ischemic stroke	NIHSS motor scores	Baseline (<12 h), Day 3, Day 30, Day 90	(1) Damage to the PLIC in the first 12 h was the best predictor of severity of motor deficits at day 90.	Puig et al. (2011)
							(2) Damage to the CS and CR at day 3 was associated with poor motor outcome at day 90.	
	Lesion (CST)	Baseline, 3 months	DWI	Ischemic stroke	FMA-UE, NIHSS motor scores	Baseline, 3 months	A weighted CST lesion load measured at the acute phase, in addition to the initial motor impairment, predicted upper limb motor outcomes (assessed by FMA-UE) at 3 months after stroke.	Feng et al. (2015)
								Doughty et al. (2016)
	CST degeneration	Baseline (≤6 h), 12 ± 6 h, 24 ± 6 h, 7 ± 2 days, 30 ± 10 days	ADC sequence	Ischemic stroke	NIHSS motor score	Baseline (≤6 h), 12 ± 6 h, 24 ± 6 h, 7 ± 2 days, 30 ± 10 days	CST ADC decreases after acute stroke in patients with poor motor outcome	DeVetten et al. (2010)
	FA asymmetry at the pons	Baseline (≤12 h), Day 3, Day 30	DTT	Ischemic stroke	NIHSS motor subscore, mRS BI MI	Baseline (≤12 h), Day 3, Day 30, Day 90, Year 2	The FA at day 30 is a predictor of motor outcome 2 years after stroke.	Puig et al. (2013)
	Intra- and interhemispheric motor connections	Baseline, 1 month, 6 months	DSI	Ischemic stroke	NIHSS FIM mRS	Baseline, 1 month, 6 months	The contralesional structural motor connections at baseline, combined with patients’ age and initial NIHSS scores, predicted functional improvement at 6 months.	Granziera et al. (2012)
Neuroimaging biomarkers (fMRI)	Motor-related brain activation	Baseline (6 ± 3 days), 12 ± 4 days, 4–6 months	fMRI BOLD signals	Ischemic stroke	A composite score using ARAT and grip strength	Baseline (6 ± 3 days), 12 ± 4 days, 4-6 months	(1) Patients with higher levels of activity in the ipsilesional M1, SMA, dPMC, and contralesional cerebellum at the acute stage predicted a good motor outcome in the chronic phase.	Rehme et al. (2015)
							(2) Patients with poor outcome showed enhanced activity in the contralesional mid-posterior insula and the ipsilesional cerebellum at the acute stage.	
	Motor-related brain activation	Baseline (10 ± 14 days), 1-4 weeks (weekly), 3 months, 6 months, 12 months	fMRI BOLD signals	Ischemic stroke	Rankin disability scale, BI, OPSS, MI, NHPT, grip strength, ARAT, timed 10 m walk.	Baseline (10 ± 14 days), 1-4 weeks (weekly), 3 months, 6 months, 12 months	A negative correlation between task-related brain activation levels and functional recovery across time was found in a number of motor-related regions.	Ward et al. (2003)
Combined approaches	SSEP+MEP	Baseline (2-5 weeks), 2 months	Time domain analysis	Ischemic and hemorrhagic stroke	FMA-UE, BI, Ashworth scale	Baseline (2-5 weeks), 2 months, 6 months, 12 months	(1) SSEPs and MEPs, in addition to clinical assessments, increased the possibility of predicting arm recovery in the long term.	Feys et al. (2000)
							(2) The combination of the motor scores and SSEPs collected at the acute phase best predicted long-term recovery outcomes.	
							(3) The combination of the three clinical assessments and MEPsat 2 months best predicted the long-term recovery outcomes.	
	The PREP algorithm (SAFE scores in MRC, MEP, and the FA asymmetry of the PLIC)	Baseline (<3 days)	DWI, MEP	Ischemic stroke	ARAT	Week 2, Week 6, Week 12, Week 26	The algorithm had positive predictive power of 88%, negative predictive power of 83%, specificity of 88%, and sensitivity of 73%.	Stinear et al. (2012)
								Stinear et al. (2017)

Abbreviations: **ADC**, **Apparent diffusion coefficient**; **ARAT**, **Action research arm test**; **BI**, **Barthel index**; **BOLD**, **Blood-oxygen-level-dependent**; **BSI**, **Brain symmetry index**; **CCT**, **Central conduction time**; **CR**, **Corona radiate**; **CS**, **Centrum semiovale**; **CST**, **Corticospinal tract**; **DSI**, **Diffusion spectrum imaging**; **DTT**, **Diffusion tensor tractography**; **DWI**, **Diffusion-weighted imaging**; **EEG**, **Electroencephalography**; **ERD**, **Event-related desynchronization**; **ERS**, **Event-related synchronization**; **FA**, **Fractional anisotropy**; **FC**, **Functional connectivity**; **FIM**, **Functional Independence Measure**; **FMA-UE**, **Fugl-Meyer Assessment-Upper Extremity**; **fMRI**, **Functional magnetic resonance imaging**; **IHI**, **Interhemispheric inhibition**; **LMPF**, **Local mean power field**; **M1**, **Primary motor cortex**; **MEG**, **Magnetoencephalography**; **MEP**, **Motor evoked potential**; **MI**, **Motricity index**; **MRC**, **Medical research council**; **mRS**, **Modified Rankin scale**; **NHPT**, **Nine hole peg test**; **NIHSS**, **National Institutes of Health Stroke Scale**; **OPSS**, **Orpington prognostic stroke scale**; **PLIC**, **Posterior limb of the internal capsule**; **PREP**, **Predict recovery potential**; **SAFE**, **Shoulder abduction and finger extension**; **SICI**, **Short-interval intracortical inhibition**; **SSEP**, **Somatosensory evoked potential**; **TMS**, **Transcranial magnetic stimulation**.

## Neurophysiological biomarkers

Electroencephalography (EEG) is a neurophysiological measure of electrical signals generated from a large population of neurons in the brain. EEG biomarkers can be yielded from either frequency-domain, power spectrum analysis, time-domain, evoked potential analysis, or time-frequency analysis of neural oscillations (Gasser and Molinari, 1996).

### Spontaneous EEG

Using frequency domain analysis, spontaneous EEG signals can be decomposed into several frequency bands: delta (1–4 Hz), theta (4–8 Hz), alpha (8–12 Hz), beta (12–30 Hz), and gamma (30–80 Hz) (Babiloni et al., 2020). The power of spontaneous neural oscillation in different frequency bands has served as the potential neural biomarker correlated with the injury and recovery from stroke. Cross-sectional studies found that the ipsilesional hemisphere is associated with an increase in low-frequency power in delta and theta bands, and a decrease in higher-frequency power in alpha and beta bands, compared with the contralesional hemisphere (Molnár et al., 2006). The alteration of cortical rhythmic activity after stroke is also functionally relevant. For example, the increase of delta power over the bilateral sensorimotor cortex (SMC) was associated with more severe neurological deficits (measured by the NIHSS), while a higher level of beta power over the ipsilesional SMC was related to a lower degree of neurological deficits (Wu et al., 2016). The redistribution of the power spectrum between lower and higher frequency oscillations can also be quantified by using ratio indices, such as delta/alpha ratio (Fanciullacci et al., 2017), theta/beta ratio (Molnár et al., 2006), and delta + theta/alpha + beta ratio (Sheorajpanday et al., 2011). The increase in lower-frequency waves and the decrease in higher-frequency waves also contributed to hemispheric asymmetry in stroke patients, using the brain symmetry index (BSI; van Putten and Tavy, 2004). The ratio index and/or the BSI have also been found to be functionally relevant cross-sectionally, as assessed by the NIHSS (van Putten and Tavy, 2004; Sheorajpanday et al., 2009; Fanciullacci et al., 2017) and the FMA upper extremity scores (FMA-UE; Saes et al., 2020).

Longitudinal studies have further confirmed that some of the cross-sectional markers are indicative of individual recovery potential after acute stroke. The BSI in theta band measured within 3 weeks after stroke was predictive for upper extremity recovery outcomes (assessed by the FMA-UE) at 26 weeks poststroke (Saes et al., 2021). In another study, the delta/alpha ratio over the ipsilesional hemisphere, as well as the BSI in delta band measured within 3 weeks after stroke, showed a positive correlation with the level of neurological deficits (assessed by the NIHSS) at 26 weeks poststroke (Saes et al., 2020).

In summary, an asymmetric hemispheric rhythmic activity can be observed with an increase in lower-frequency activities and a decrease in higher-frequency activities over the ipsilesional hemisphere rather than over the contralesional hemisphere. The redistribution in the power spectrum of spontaneous EEG observed at the acute stroke phase is also an indication of recovery three to 6 months after stroke (Saes et al., 2020; Saes et al., 2021). An increase in lower-frequency activities (e.g., delta and theta bands) and a decrease in higher-frequency activities (e.g., alpha and beta bands) after acute stroke indicate an unfavorable recovery outcome three to 6 months after stroke.

### Evoked potential

EEG demonstrates excellent temporal resolution at millisecond level; however, the frequency domain analysis of EEG alone cannot utilize any temporal information as the continuous data are merged across the time domain (Gasser and Molinari, 1996). Event-related potential (ERP) is a time- and phase-locked evoked response to a variety of stimuli or events, thus it gives more context-specific information about how the brain processes afferent signals and makes behavioral responses (Müller-Putz, 2020). Therefore, ERP has the potential to be used as a biomarker in the prediction of poststroke functional recovery.

Somatosensory evoked potential (SSEP) is a form of EEG evoked potential elicited by somatosensory stimuli, such as electrical stimulation to a peripheral nerve. SSEP is believed to be associated with the activation of the somatosensory cortex along with somatosensory ascending volley and is therefore a proxy for functional integrity of the somatosensory pathway (Guerreiro and Ehrenberg, 1982). SSEP has demonstrated prognostic values of functional recovery after stroke. Stroke patients with or without SSEP response at baseline showed a significant difference in the amount of recovery of the hemiparetic arm at 6 and 12 months poststroke (Feys et al., 2000), and the pathological change in the morphology of SSEP was associated with a higher level of disability (mRS scores ≥5) at 6 months poststroke (Su et al., 2010).

### Time-frequency oscillatory activities

Time-frequency analysis decomposes EEG signals into both time and frequency domains, therefore reserving the temporal evolution of neural dynamics across different frequency bands (Pfurtscheller, 2001). Event-related desynchronization (ERD) is typically elicited by neuronal activation and can therefore be regarded as a marker of cortical activation and excitability, while event-related synchronization (ERS) can be interpreted as a deactivated cortical region (Pfurtscheller, 2001). Movement-related ERD/ERS has been investigated in stroke research. An ERD in both mu and beta bands can be observed during movement preparation and execution, while an ERS in beta band can be found after the termination of movement (Pfurtscheller and Lopes Da Silva, 1999). A reduction of movement-related beta ERD over the ipsilesional sensorimotor cortex (SMC) during hemiparetic hand movement was found in acute (Tangwiriyasakul et al., 2014), subacute (Bartur et al., 2019; Tang et al., 2020), and chronic stroke patients (Rossiter et al., 2014). In addition, the level of movement-related ERD was associated with the level of upper limb motor impairment (Rossiter et al., 2014; Bartur et al., 2019; Tang et al., 2020), functional activity (Tang et al., 2020), and the lesion volume of structural damage caused by stroke (Bartur et al., 2019). Although the findings were based on cross-sectional experiments, they also indicate the potential predictive value of ERD/ERS.

Tangwiriyasakul et al. (2014) investigated the longitudinal change of ERD patterns in the bilateral hemisphere accompanying stroke recovery. An increase in movement-related ERD over the ipsilesional hemisphere and a decrease in movement-related ERD over the contralesional hemisphere were observed across time from one to 4 weeks after stroke onset (Tangwiriyasakul et al., 2014). In another longitudinal study, postmovement beta ERS (16–30 Hz) peak amplitude over the ipsilesional SMC at the acute phase, predicted the upper limb recovery outcomes in FMA-UE 12 weeks after stroke (Tang et al., 2020). ERD/ERS can also be induced by observation of movement. Zhang et al. (2022) found that mirror visual feedback-induced high beta ERD (21–30 Hz) over the ipsilesional SMC was associated with motor training-induced improvement assessed by FMA-UE and ARAT in chronic stroke patients (Zhang et al., 2022). However, ERD/ERS induced by movement observation has not been implicated in the prediction of functional recovery after acute stroke, and awaits further investigation (Zhang et al., 2022).

To sum up, the findings from time-frequency EEG analyses suggest a predictive role of sensorimotor beta oscillation in poststroke functional recovery, specifically in the motor domain. However, in most studies, ERD/ERS is triggered with the voluntary movement of the hemiparetic hand. The potential predictive role of ERD/ERS induced by other forms of stimuli or events needs to be further investigated in stroke patients.

### EEG connectivity

The connectivity measured by EEG refers to the relationship between two or more scalp EEG sensors (Guevara and Corsi-Cabrera, 1996). There are plenty of EEG connectivity parameters that have been used in clinical studies, among which, coherence, which measures the similarity between two signals in the frequency domain, has been applied in stroke research with prognostic values (Cassidy et al., 2020). The EEG connectivity provides unique insights into the dynamic intra-/interhemispheric interactions; therefore, it can further facilitate the conceptualization of recovery models poststroke from a brain network perspective.

According to two cross-sectional studies, high beta (20–30 Hz) coherence between the ipsilesional M1 and the premotor cortex positively correlated with the arm motor recovery as measured by the FMA-UE (Wu et al., 2015), while delta (1–3 Hz) coherence between the ipsilesional and contralesional M1 inversely correlated with the arm function as measured by the FMA-UE (Cassidy et al., 2020). Longitudinally, a decrease in delta coherence between the bilateral M1 over the first 3 months poststroke correlated with arm motor recovery in the FMA-UE (Cassidy et al., 2020). In another study with chronic stroke patients, a greater degree of high beta coherence between the M1 and premotor cortex over the ipsilesional hemisphere was positively correlated with motor gains in the FMA-UE after 1 month of intensive motor training, while a greater degree of high beta coherence between the M1 and parietal cortex over the ipsilesional hemisphere negatively correlated with motor gains in response to training (Wu et al., 2015). Taken together, the results indicate that the prediction of stroke recovery using EEG connectivity outcomes is likely influenced by frequency bands, brain regions, and time after stroke onset.

### Single-pulse induced motor evoked potential (MEP)

Transcranial magnetic stimulation (TMS) studies revealed the potential values of using MEP for functional prognosis. MEP is a marker to measure the functional integrity and excitability of the corticospinal tract (CST; Hallett, 2007). According to a seminal study, the presence of MEP evoked by the ipsilesional M1 stimulation in the acute stroke stage was associated with good recovery at 6 months after stroke, defined as a BI ≥ 60 and/or strength index of the paretic limb muscles in the medical research council (MRC) scale ≥4, while the absence of MEP evoked by the ipsilesional M1 stimulation was related to poor recovery (measured by the BI or the MRC scale), with sensitivity from 77.1 to 87.1% and specificity from 81.0% to 85.7% (Escudero et al., 1998). MEP studies mainly focused on upper extremity motor recovery after stroke and their results suggest that the presence of MEP evoked by the ipsilesional M1 stimulation at the acute phase indicate a sign of good recovery (Escudero et al., 1998); however, the absence of the MEP evoked by the ipsilesional M1 stimulation at the acute phase does not imply poor recovery (Powell et al., 2019).

### Paired-pulse TMS outcomes

Paired-pulse TMS paradigms have been used to investigate the intracortical or interhemispheric facilitation/inhibition after stroke. In the paired-pulse paradigms, two consecutive pulses, i.e., a conditioning stimulus (CS) and a testing stimulus (TS), are delivered to one or over two different cortical regions. Depending on the intensity of the CS as well as the interval between the CS and TS, paired-pulse TMS can result in either facilitatory or inhibitory influence on the induced MEP. In a longitudinal study by Manganotti et al. (2008), the change of the short-interval intracortical inhibition (SICI), i.e., subthreshold CS and suprathreshold TS to the unaffected M1, with an interstimulus interval of 2–4 ms, was correlated with upper extremity motor improvement (assessed by the Lindmark scale) from the acute phase (day 5–8) to 3-month poststroke, suggesting that the increase of motor inhibition over the contralesional M1 mirrored the motor improvement poststroke. Subgroup analysis revealed that the longitudinal correlation only existed in patients with a lower level of disability at baseline (BI ≥ 60), but not in patients with a higher level of disability. However, when using an intracortical facilitation (ICF) paradigm (subthreshold CS and suprathreshold TS to each M1, with an interstimulus interval of 7–15 ms), no significant correlation was observed (Manganotti et al., 2008). In another longitudinal study (Xu et al., 2019) using an interhemispheric inhibition (IHI) paradigm (subthreshold CS to the contralesional M1 and suprathreshold TS to the ipsilesional M1, with an interval of 10 ms), stroke patients at the acute and subacute phases demonstrated a normal level of IHI prior to movement onset, in comparison to their healthy counterparts. However, stroke patients at the chronic phase showed abnormal IHI prior to movement onset—the contralesional M1 remained inhibitory to the ipsilesional M1 before paretic hand movement. The longitudinal emergence of abnormal premovement IHI from the acute to chronic phases was negatively correlated with the improvement of hand functions in a finger individualization task. Therefore, this study concluded that the existence of an inhibitory flow from the contralesional to the ipsilesional M1 may not be the cause of poor recovery poststroke (Xu et al., 2019).

### Concurrent TMS-EEG outcomes

TMS-evoked EEG potential (TEP), by its nature, is a form of ERP, reflecting the brain’s responsivity to an external stimulus using a single TMS pulse. Besides, TMS-EEG outcomes can also be analyzed in the time-frequency domain to reflect the ongoing neural oscillation perturbated by TMS (Tremblay et al., 2019). Cross-sectionally, the ipsilesional hemisphere showed a simplified focal response to TMS pulses, with a smaller number of deflections and a higher amplitude in the TEP curve, compared with the contralesional hemisphere and healthy controls (Tscherpel et al., 2020). After considering the initial deficits and the ipsilesional MEP in a multivariate regression analysis, TMS-EEG outcomes are significant variables in the prediction model with a composite recovery score of the NIHSS, ARAT, and grip strength measured by a vigorimeter as the dependent variable (Tscherpel et al., 2020). The results indicate a complementary role of using TMS-EEG outcomes in predicting poststroke motor recovery, in addition to the ipsilesional MEP and the initial motor deficits.

Succinctly, the redistribution of rhythmic powers (spontaneous EEG powers at resting-state and ERD/ERS), pathological alterations of cortical evoked potentials (MEP, SSEP, and TEP), as well as change of the intra-/interhemispheric coherent activities represent distinct forms of functional disturbance of hemispheric activities after acute stroke. The evidence from neurophysiological studies largely supports that a greater level of initial interhemispheric imbalance poststroke predicts poorer motor and functional outcomes. Besides, paired-pulse TMS studies highlight the role of contralesional M1, especially for its intracortical inhibition, in monitoring poststroke motor recovery over time.

## Neuroimaging biomarkers

### Structural MRI

Structural MRI biomarkers provide straightforward information about the nature of stroke injury that may be relevant to functional prognosis (Jiang et al., 2010). Routine MRI techniques for acute stroke include T1- and T2-weighted imaging, diffusion-weighted imaging (DWI), etc. (Nanjundaswamy et al., 2011). In addition, diffusion tension imaging (DTI) is often used for measuring microstructural alteration of structural connections *via* white matter (Puig et al., 2017). No consistent correlation was found between global lesion volume and recovery outcomes (Schiemanck et al., 2006; Rehme et al., 2015); however, lesion to certain functionally relevant anatomical structures could determine, at least partially, the amount of functional recovery after stroke (Puig et al., 2017). For example, damage to the posterior limb of the internal capsule, which contains the corticospinal descending fibers, could best predict the severity of neurological deficits (assessed by the NIHSS) 3 months after stroke (Puig et al., 2011). Quantitative measures of CST show prognostic values as well: a weighted CST lesion load in DWI, measured at the acute phase, in addition to the initial motor impairment, predicted motor recovery (measured by the FMA-UE) at 3 months after stroke (Feng et al., 2015; Doughty et al., 2016). Similarly, a greater extent of CST degeneration, as revealed by its apparent diffusion coefficient (ADC) at the acute phase, was associated with a more severe neurological deficit at 3 months after stroke (DeVetten et al., 2010). The laterality index of the fractional anisotropy (FA) obtained from DTI is also commonly used in stroke recovery prediction—the level of FA asymmetry at the pons measured 1-month poststroke was found to be predictive of motor recovery 2 years after stroke, as assessed by the motricity index (Puig et al., 2013).

Apart from the CST, the intra- and interhemispheric corticocortical connection of the motor system (the M1, premotor, and supplementary motor area [SMA]) also shows a prognostic value (Salvalaggio et al., 2020). For example, using diffusion spectrum imaging MRI, generalized fractional anisotropy of both intra- and interhemispheric motor connections at the contralesional hemisphere measured at the acute phase, combined with age and the initial severity of stroke, could explain 96% of the variance of the severity of neurological deficits (measured by the NIHSS) at 6 months after stroke (Granziera et al., 2012). This evidence indicates the compensatory role of structural motor connections over the contralesional hemisphere in motor recovery poststroke.

### Functional MRI

Functional MRI (fMRI) provides an indirect measure of neuronal activity by detecting brain oxygenation and hemodynamics (Crofts et al., 2020). The level of the ipsilesional M1 activation during paretic hand movements, measured by fMRI, is strongly correlated with the level of upper extremity motor impairment, according to two meta-analyses (Rehme et al., 2012; Favre et al., 2014). In addition, better motor performance in patients with stroke is found to be related to a higher level of activation over the ipsilesional premotor cortex, pre-SMA, as well as the contralesional premotor cortex and cerebellum (Rehme et al., 2012). Therefore, functional activation of the motor-related system has been assessed as a form of imaging biomarker for prognostic prediction of poststroke recovery (Ward et al., 2003). For instance, neural activation during handgrip of the paretic side measured at the acute phase could predict motor outcomes at four to 6 months after stroke (Rehme et al., 2015). Patients with a good outcome (assessed by grip strength and the ARAT) showed a higher level of activation over the ipsilesional M1, SMA, and dorsal premotor cortex, and the contralesional cerebellum at the acute stroke phase (6 ± 3 days), compared with those with a poor outcome. However, results from a subsequent fMRI measure at 12 ± 4 days were not predictive (Rehme et al., 2015). Interestingly, other longitudinal fMRI studies demonstrated different forms of neuroplastic changes in patients after stroke (Ward et al., 2003; Hamzei et al., 2006). In acute stroke patients, overactivation in the primary and secondary motor areas was found initially. The movement-related brain activation started to reduce gradually over time, and a negative correlation between the change of brain activation levels in the cortical motor areas and the amount of functional recovery was reported in ischemic stroke patients without M1 lesion involvement (Ward et al., 2003). Research also indicates the neuroplastic change after stroke differs in patients with different levels of structural lesions. For example, a study with chronic stroke patients showed that patients with evocable ipsilesional MEP and intact hand area of M1 showed a reduction of the ipsilesional SMC activation along with recovery, while patients with a lesion involving the M1 or absent ipsilesional MEP showed an increase in the ipsilesional SMC activity accompanying recovery (Hamzei et al., 2006). These findings suggest that the structural and functional integrity of the CST may mediate the recovery patterns of brain activations after stroke.

Taken together, structural damage to CST as well as neuroimaging biomarkers of motor connections could potentially predict motor recovery after stroke. The level of activation of the motor system over the ipsilesional hemisphere during motor execution may also be indicative of the potential for recovery (Rehme et al., 2012; Favre et al., 2014). However, the structural lesion is likely to play a mediating role in brain reorganization, leading to different recovery and brain activation patterns (Ward et al., 2003; Hamzei et al., 2006; Rehme et al., 2015).

## Combined approaches

A combination of different neurophysiological and neuroimaging biomarkers in the prediction of stroke prognosis has been investigated in a few studies (Feys et al., 2000; Lee et al., 2010; Stinear et al., 2012; Lee et al., 2015; Stinear et al., 2017). For example, MEP is sometimes used together with SSEP for poststroke prediction—this approach offers a measure of both somatosensory ascending and motor descending pathways, which is related to poststroke sensorimotor functions. The combination of the initial status of both MEP and SSEP showed improved predictive values of neurological deficits (Lee et al., 2010), arm motor functions (Feys et al., 2000), and balance functions (Lee et al., 2015) at the subacute/chronic stage after stroke.

The Predict Recovery Potential (PREP) algorithm is an established biomarker-based approach for predicting individual poststroke upper extremity recovery outcomes, which uses a combination of MRI, MEP, and motor behavioral tests (Stinear et al., 2012). The initial algorithm was validated in a sample of 40 patients with stroke (Stinear et al., 2012) and later in a large sample of 192 patients (Stinear et al., 2017). The biomarkers selected in this prediction algorithm are MEP and FA asymmetry at the posterior limb of the internal capsule, representing the functional and structural integrity of the CST, respectively. With biomarkers collected at the acute stage and the initial motor deficits, the algorithm had an overall accuracy of 80% in prognostic prediction of outcome at 12 weeks post-stroke (Stinear et al., 2017).

## Biomarkers collected using molecular neuroscience approaches

A wide variety of molecular, genetic, and epigenetic biomarkers have been used to predict stroke prognosis. A list of biomarkers in this category is presented in Table 2. Molecular, genetic, and epigenetic biomarkers can be classified according to their physiological function and nature in hemostasis, inflammation, tissue remodeling, apoptosis, oxidative stress, infection, metabolism, brain-derived, neuroendocrine, and cardiac biomarkers (Maas and Furie, 2009).

**TABLE 2:** Molecular, genetic and epigenetic biomarkers for stroke prognosis.

Type of biomarker	Biomarker	Measurement time points (biomarker)	Matrix	Analytical methods	Type of stroke	Functional outcomes	Measurement time points (functional outcome)	Main findings	References
Brain-derived biomarkers (neuronal and glial biomarkers)	S100B	Baseline (<24 h)	Peripheral blood, cord blood, saliva, urine, CSF, amniotic fluid, and milk	Immunoradiometric assay, immunolimumetric assay, mass spectroscopy, Western blot, ELISA, electrochemiluminiscence, RT-PCR	Ischemic stroke	NIHSS	Baseline, 3 months	High concentrations were associated with poor outcomes.	Abraha et al. (1997)
									Yardan et al. (2011)
									Michetti et al. (2012)
									Kamtchum-Tatuene and Jickling (2019)
									Rahmati et al. (2020)
	Progranulin	Baseline (<24 h)	Serum	ELISA	Ischemic stroke	mRS	Baseline, 6 months	High levels were associated with poor functional outcomes.	Xie et al. (2016)
	NfL^a^	Baseline (<24 h), 3 months	CSF and serum	Homebrew assay on the single-molecule array (SiMoA) platform (Quanterix)	Ischemic stroke	NIHSS, mRS	Baseline, Discharge, 3 months	High levels associated with poor functional outcomes.	Wang et al. (2020b)
	GFAP	Baseline (<24 h)	CSF and serum	ELISA and quantitative proteomics analysis by 4D label-free quantitative method-diaPASEF (4DLFQ)	Ischemic stroke	NIHSS	Baseline, 1 year	High levels predicted poor functional outcome and mortality.	Liu and Geng (2018)
									Amalia (2021)
									Montellano et al. (2021)
	NSE	(a) Baseline (<6 h and <48 h)	(a), (b), and (c) serum, (d) CSF	(a), (b), (c), and (d) ELISA	(a) and (d) Ischemic and hemorrhagic stroke, (b) and (c) Ischemic stroke	(a), (b), and (c) NIHSS, (c) and (d) mRS	(a) Baseline, 28 days,	(a), (b), (c), and (d) higher concentrations were associated to higher NIHSS, (c) and (d) Higher concentrations were correlated to higher mRS.	(a) Shash et al. (2021)
		(b) Baseline (12–48 h)					(b) Baseline, 60 days		(b) González-García et al. (2012)
		(c) Baseline (shortly after symptom onset)					(c) Baseline, 90 days		(c) Kurakina et al. (2021)
		(d) Baseline (at admission)					(d) Baseline, 14 days		(d) Nasution and Bangun (2022)
Neuroendocrine biomarkers	Copeptin	Baseline (at admission)	Serum	Sandwich immunoluminometric assay	Ischemic stroke	mRS	Baseline, 3 months, 1 year	High levels were associated with poor functional outcome within 3 months or 1 year after the stroke event.	Liu and Geng (2018)
									Amalia (2021)
									Montellano et al. (2021)
	Leptin/adiponectin	Baseline (at admission)	(a) and (b) serum	(a) and (b) ELISA	(a) and (b) ischemic stroke	(a) mRS,	(a) and (b) Baseline, 3 months	(a) High serum levels of leptin and leptin/adiponectin ratio >1.16 predicted poor outcomes.	(a) Carbone et al. (2015)
						(b) NIHSS		(b) High levels of adiponectin predicted poor outcomes.	(b) Wang et al. (2019b)
Hemostasis biomarkers	Fibrinogen^a^	Baseline (on the day of symptom onset), 3 months	Serum	Coagulometric method (Automated Sysmex Cs 2000 analyzer)	Ischemic stroke	NIHSS	Baseline, 3 months	High levels of fibrinogen predicted poor outcomes.	Hou et al. (2021)
	hFABP	Baseline (at admission)	Plasma	ELISA	Ischemic stroke	mRS	Baseline, 3 months	High plasma concentration was associated to poor outcomes.	Park et al. (2013)
	D-dimer	(a) Baseline (at admission)	(a) and (b) Plasma	(a) Latex-enhanced immunoturbidimetric test for the quantitative determination of cross-linked fibrin degradation products in plasma	Ischemic stroke	(a) and (b) mRS	(a) Baseline, 1 month	(a) High concentrations of D-dimer in plasma correlated with poor outcomes.	(a) Abd-Elhamid et al. (2016)
		(b) Baseline (at admission)		(b) Not reported			(b) Baseline 3 months, 6 months, 12 months	(b) A positive and dose-dependent relationship between D-dimer levels and poor clinical outcome was observed.	(b) Wang et al. (2020a)
	Fibulin-5	(a) Baseline (<1 hour)	(a) and (b) Serum	(a) and (b) ELISA	(a) Ischemic and hemorrhagic stroke	(a) and (b) mRS	(a) and (b) Baseline, 3 months	(a) and (b) High concentration of fibulin-5 predicted poor outcomes.	(a) Elshony et al. (2021)
		(b) Baseline (<3 days)			(b) Hemorrhagic stroke				(b) Hu et al. (2016)
Inflammation biomarkers	mHLA-DR	Baseline (<36 h)	Plasma	FACS	Ischemic stroke	mRS	Baseline, 3 months	Low levels of mHLA-DR were associated with poor clinical outcome and shorter survival time.	Mengel et al. (2019)
	IL-6^a^	(a) Baseline (<36 h)	(a) and (c) Plasma	(a) IMMULITE semi-automatic chemiluminescent immunoassay	(a–d) Ischemic stroke	(a) mRS	(a), (b) and (d) Baseline, 3 months,	(a–d) High levels were associated with poor function outcomes.	(a) Mengel et al. (2019)
		(b) Baseline (4, 8, 12, 24, 48, and 72 h, Day 7, Day 12, 3 months, 1 year)		(b) high sensitivity quantitative sandwich enzyme immunoassays		(b) European stroke scale and BI			(b) Waje-Andreassen et al. (2005)
		(c) Baseline (6–24 h), Day 5	(b) and (d) Serum	(c) ELISA		(c) mRS and NIHSS	(c) Baseline, 3 months, 1 year		(c) Shaafi et al. (2014)
		(d) Baseline (<24 h), 3 months		(d) ELISA		(d) mRS and NIHSS			(d) Aref et al. (2020)
	CRP^a^	Baseline (<24 h)	Plasma	Enzyme linked immunosorbent assay	Ischemic stroke	mRS	Baseline, 3 months	High levels were associated with poor outcomes.	Gu et al. (2022)
	MBL	(a) Baseline (<72 h)	(a) and (b) Serum	(a) Sandwich-ELISA	(a) and (b) Ischemic stroke	(a) and (b) mRS	(a) and (b) Baseline, 3 months	(a) Low levels were associated to poor outcomes.	(a) Osthoff et al. (2011)
		(b) Baseline (<24 h)		(b) ELISA				(b) High levels predicted poor outcomes.	(b) Zhang et al. (2015)
	25-(OH)D	(a) Baseline (<24 h)	(a) and (b) Serum	(a) E601 modular	(a) and (b) Ischemic stroke	(a) NIHSS,	(a) Baseline, Discharge (mean hospital stay of 31 days)	(a) Vitamin D deficiency was negatively correlated to increasing severity of stroke,	(a) Wang et al. (2014b)
		(b) Baseline (<48 h)		(b) Competitive chemiluminescent immunoassay on Elecsys 2010		(b) and (c) mRS	(b) Baseline, 3 months	(b) Favorable outcomes were associated with higher levels of serum 25-OHD.	(b) Tu et al. (2014)
	YKL-40	(a) Baseline (48–72 h)	(a) and (b) Plasma	(a) and (b) ELISA	(a) Ischemic stroke,	(a) and (b) mRS	(a) Baseline, 3 months	(a) High levels associated with poor functional outcomes and stroke severity.	(a) Park et al. (2012)
		(b) Baseline (<24 h)			(b) Ischemic stroke and transient ischemic attack		(b) Baseline, 3 months, 1-5 years annually	(b) High levels were associated with increased risks of poor functional outcomes.	(b) Li et al. (2022)
Oxidative stress biomarkers	Superoxide dismutase^a^	Baseline (<24 h), 72 h, Day 5, Day 10	Serum	Chemiluminescence method	Ischemic stroke	SSS	Baseline, Day 5, Day 10	Neurological deficits were negatively correlated with SOD activity.	Spranger et al. (1997)
	MDA	Baseline (<24 h)	Serum	Enzymatic peroxidation reaction with thiobarbituric acid	Ischemic stroke	NIHSS, mRS	Baseline, 3 months	An elevated serum MDA level at admission was independently associated with a poor functional outcomes 3 months after stroke.	Elsayed et al. (2020)
Metabolic biomarkers	RBP4	Baseline (<48 h)	Plasma	ELISA	Ischemic stroke	NIHSS	Baseline, 3 months	High RBP4 levels were associated with increased risk of acute ischemic stroke and poor functional outcomes.	Liu and Che (2019)
	HbA1c	Baseline (<24 h)	NA	NA	Ischemic stroke	NIHSS, mRS	Baseline, 3 months	High levels are associated with poor functional outcomes in acute ischemic stroke.	Wang et al. (2019a)
	MMP-9	Baseline (<24 h)	Serum	ELISA	Ischemic stroke	NIHSS, mRS	Baseline, 3 months	A higher serum MMP-9 level in acute ischemic stroke was associated with an increased risk of disability.	Zhong et al. (2017)
Infection biomarkers	LBP	Baseline (<1 day)	Plasma	NA	Ischemic stroke	mRS	Baseline, 3 months	High levels of LBP were associated with poor outcomes.	Mengel et al. (2019)

Abbreviations: **25-(OH)D**, **25-hydroxyvitamin D**; **ADAMTS13**, **A disintegrin and metalloprotease with thrombospodin type 1 motif 13**; **BNIS**, **Barrow Neurological Institute Screen for Higher Cerebral Functions**; **CRP**, **C-reactive protein**; **CSF**, **Cerebrospinal fluid**; **FACS**, **Fluorescence activated cell sorting**; **GFAP**, **Glial fibrillary-associated protein**; **HbA1c**, **Glycated hemoglobin**; **hFABP**, **Heart-type fatty acid binding protein**; **IL-6**, **Interleukin-6**; **LBP**, **Lipopolysaccharide-binding protein**; **MBL**, **Manose-binding lectin**; **MDA**, **Malondialdehyde**; **mHLA-DR**, **Monocytic HLA-DR**; **MMP-9**, **Matrix metalloproteinase-9**; **MR-proANP**, **Mid-regional pro-atrial natriuretic peptide**; **mRS**, **modified Ranking Scale**; **NA**, **Not available**; **NfL**, **Neurofilament light chain**; **NIHSS**, **National Institutes of Health Stroke Scale**; **NSE**, **Neuron-specific enolase**; **NT-proBNP**, **N-terminal pro-B-type natriuretic peptide**; **RBP4**, **Retinol-binding protein 4**; **SSS**, **Scandinavian Stroke Scale. TNF-α**, **Tumor necrosis factor α**.

a Shows the biomarkers that have been repeatedly measured at follow-up.

## Brain-derived biomarkers (neuronal and glial biomarkers)

### Serum calcium-binding protein (S100B)

The S100B is a calcium-binding peptide abundantly found within glial cells and is used as a marker of glial activation and/or death in several neurological disorders (Yardan et al., 2011; Kamtchum-Tatuene and Jickling, 2019; Vuuren et al., 2022). High levels of S100B in cerebrospinal fluids (CSF) and the peripheral circulation have been associated with pathological injury and neuronal damage, and reliably predict injury severity (Yardan et al., 2011; Vuuren et al., 2022). In the course of metabolic injury associated with oxygen, serum, and glucose deprivation, glial cells respond by secreting S100B, and high concentrations of S100B lead to neuronal death *via* nitric oxygen release from astrocytes (Yardan et al., 2011). The prolonged presence of S100B in serum is an indication of its continuous release from affected tissue (Yardan et al., 2011). In ischemic stroke, high levels of S100B have been associated with the degree of systemic inflammation disregarding the size of the infarction (Michetti et al., 2012), therefore S100B has been proposed as a potential biomarker to predict recovery outcomes after stroke (Rahmati et al., 2020).

High levels of S100B in serum could significantly predict the level of neurological disability at 3 months after ischemic stroke, measured by the BI, mRS, and Lindley score (Abraha et al., 1997; Abd-Elhamid et al., 2016). In another study, S100B was measured in blood 3 months after ischemic stroke (Rahmati et al., 2020) and a significant correlation between high levels of S100B and worse prognosis (measured by the mRS and NIHSS) was observed (Rahmati et al., 2020). Similarly, high concentrations of S100B (blood samples taken 24 h after symptom onset) detected in serum by ELISA, were associated with more severe neurological deficits (NIHSS>10) after ischemic stroke (Altintas Kadirhan et al., 2021). These findings suggest that S100B could be a powerful prognostic biomarker in ischemic stroke.

### Progranulin

Progranulin, a 68.5 kDa cysteine-rich secreted glycoprotein, is a multipotent growth factor also known as proetithelin 1, acrogranin 2, PC cell-derived growth factor 3, and granulin-epithelin precursor 4 (Xie et al., 2016; Abella et al., 2017). Progranulin is involved in several biological and pathological processes, including cell growth, tumorigenesis, wound healing, inflammation, and insulin resistance (Abella et al., 2017). Progranulin deficiency and overexpression have been associated with neurodegeneration and the onset of different types of cancer, respectively (Abella et al., 2017). This growth factor is primarily found in mature neurons and microglia, in which its expression is upregulated in response to injury and inflammation (Alexander et al., 2019).

The relationship between progranulin levels in serum and adverse functional outcomes after stroke has been studied. The levels of progranulin in fasting blood samples taken on the first morning after admission were analyzed by ELISA. A higher level of progranulin was correlated with a higher level of physical disability (measured by mRS) 6 months after stroke (Xie et al., 2016).

### Neurofilament light chain (NfL)

NfL is a cytoskeletal protein whose expression is limited to neurons and has emerged as a neuronal injury biomarker (Barro et al., 2020). The neuronal cytoskeleton is composed of neurofilaments consisting of three subunits, namely neurofilament light, neurofilament medium, and neurofilament heavy chains (Yuan et al., 2012). The NfL, together with the medium and heavy subunits, is one of the scaffolding proteins implicated in axonal injury that gets released into the extracellular matrix following axonal damage (Wang P. et al., 2020).

In patients with small vessel disease, NfL levels remained elevated for at least 3 months post-stroke (Wang P. et al., 2020). Measured in the first 24 h after admission, high concentrations of NfL can predict poor outcomes, as observed by the positive correlation between mRS, NIHSS scores, and serum levels of NfL (Wang and Wei, 2009). The advantage of NfL as a biomarker of neuronal damage is that it does not only predict prognosis if measured 24 h after admission (Wang P. et al., 2020), but it can also be used to quantify neuronal damage in stroke to predict recovery progress (Barro et al., 2020). NfL levels are not specific to stroke as they can also be influenced by physiological processes such as the body mass index and disease states (e.g., diabetes and hypertension; (Barro et al., 2020). Therefore, studies to validate NfL as a prognostic biomarker should consider the factors that can also affect NfL levels.

### Glial fibrillary acidic protein (GFAP)

GFAP is a monomeric filament protein found as the main component of the cytoskeleton of mature astrocytes in the central nervous system (Amalia, 2021). Astrocytes react in response to injury or ischemic processes to maintain cell homeostasis through complex response mechanisms known as astrocytosis (Liu and Geng, 2018; Cole and Einstein, 2019; Amalia, 2021). In ischemic stroke, high levels of GFAP detected in CSF and serum in samples taken within 24 h after symptom onset were correlated to higher NIHSS scores (Amalia, 2021). GFAP levels peak after 48–74 h after symptom onset with a positive relationship between elevated GFAP levels, the severity of neurological deficits, and the extent of the brain damage (Amalia, 2021). Higher GFAP levels in serum predicted poorer outcomes according to the NIHSS at 1-year follow-up poststroke (Liu and Geng, 2018).

### Neuron-specific enolase (NSE)

NSE, also known as the γγ-isoenzyme of the glycolytic enzyme enolase, gets released into the extracellular matrix as a result of neuronal injury and death, and can be detected in blood shortly thereafter (Kurakina et al., 2021; Shash et al., 2021). NSE has been used as a biomarker to assess the extent of structural and functional impairment in the central nervous system (Kurakina et al., 2021). Although no consensus has been reached on the correlation between NSE level and the severity of neurological symptoms in stroke (Kurakina et al., 2021), recent evidence supports its usefulness as a prognosis biomarker in stroke. NSE serum levels measured in samples taken shortly after symptom onset were high in patients with higher NIHSS scores measured 28 days after stroke (Shash et al., 2021). In a study following up patients for 90 days, high NSE levels were positively correlated with higher scores in the NIHSS and mRS (Kurakina et al., 2021). A similar trend was observed in ischemic stroke patients in a study assessing functional recovery 60 days after stroke using the NIHSS (González-García et al., 2012). NSE levels were measured in blood samples taken between 12 and 48 h after symptom onset, and NSE has positively correlated to the neurological outcome in stroke patients (González-García et al., 2012). In CSF and blood samples taken from both ischemic and hemorrhagic stroke patients, NSE was identified between four and 8 hours after symptom onset (Nasution and Bangun, 2022). NSE levels positively correlated with functional outcomes assessed using the NIHSS and mRS at 14 days post-stroke (Nasution and Bangun, 2022). In blood samples taken within the first 48 h after ischemic stroke, high NSE levels were correlated with the severity of neurological symptoms, assessed by the NIHSS (Kurakina et al., 2021). On the other hand, low levels of NSE were associated with good motor function outcomes 2 weeks after symptom onset, suggesting that NSE is a good predictor of motor recovery, measured by the Rivermead mobility index (Kurakina et al., 2021).

## Neuroendocrine biomarkers

### Copeptin

Copeptin, the C-terminal portion of pro-vasopressin, is a neurohypophysis hormone released in an equimolar ratio to vasopressin mirroring the levels of vasopressin (Zhang et al., 2013; Xu et al., 2017; Kamtchum-Tatuene and Jickling, 2019). In stroke, vasopressin release is driven by cerebral edema, volume overload, and hyponatremia, and copeptin increases in bacterial infections and febrile conditions (Zhang et al., 2013). Hypoxemia can lead to abnormal renal water excretion, which is most likely mediated *via* vasopressin release (Zhang et al., 2013; Wang C.-W. et al., 2014).

Several studies place copeptin as one of the most promising strong and independent biomarkers in ischemic stroke that predicts functional outcomes (Katan et al., 2009; De Marchis et al., 2013; Dong et al., 2013; Tu et al., 2013; Zhang et al., 2013; Wang C.-W. et al., 2014; Xu et al., 2017). A meta-analysis that evaluated the role of copeptin in prognostic prediction after ischemic stroke revealed that high levels of copeptin (tenfold increase) were associated with poor functional outcomes within 3 months or 1 year after stroke (Xu et al., 2017). Blood samples of the studies included in the meta-analysis were taken on the first day of admission (Xu et al., 2017). Furthermore, copeptin improved the prognostic value of the NIHSS compared to other prognostic biomarkers (Zhang et al., 2013).

### Leptin/adiponectin

Leptin, a 16 kDa protein secreted by the adipose tissue, is responsible for the regulation of food intake, non-shivering thermogenesis, reproduction, hemostasis, angiogenesis, regulation of arterial pressure, and neuroendocrine and immune functions (Zhao et al., 2021). Adiponectin, a 30 kDa glycoprotein expressed in adipose tissue, regulates the body’s sensitization to insulin (Wang Z. et al., 2019; Zhao et al., 2021). Low concentrations of adiponectin have been linked to disorders such as metabolic syndrome, atherosclerosis, hypertension, and cardiovascular disease, while high concentrations have been linked to increased risk for ischemic stroke (Wang Z. et al., 2019). Leptin functions as a neuroprotective agent against ischemic neuronal injury (Zou et al., 2020), while it has been suggested that adiponectin increases to counteract the systemic inflammation conditions observed in ischemic stroke (Wang Z. et al., 2019). A peak in serum levels of leptin and leptin/adiponectin ratio >1.16 has been observed 1 day after ischemic stroke and predicted poor functional outcomes measured using mRS after 3 months (Carbone et al., 2015; Kamtchum-Tatuene and Jickling, 2019). Also, high levels of adiponectin have been independently associated with poor prognosis (mRS scores 3–6) in ischemic stroke patients in a 3-month follow-up study (Wang Z. et al., 2019).

## Hemostasis biomarkers

### Fibrinogen

Fibrinogen is an inflammation-related protein that participates in thrombokinesis, platelet aggregation, blood viscosity, thrombus formation, and fibrin formation (Kim and Lee, 2014; Zhai et al., 2022). Fibrinogen is secreted in the liver and plays a role in cardiovascular risk through the regulation of coagulation and inflammation pathways (Hou et al., 2021). High concentrations of this hemostatic biomarker are directly associated with a higher incidence of ischemic stroke (Kim and Lee, 2014). In blood samples collected shortly after the onset of ischemic stroke, the levels of fibrinogen were found to be high (Kim and Lee, 2014; Sale et al., 2016; Peycheva et al., 2021). A negative correlation between high fibrinogen and improved outcomes measured by the NIHSS in ischemic stroke patients was reported, suggesting that high fibrinogen levels were an indicative biomarker of poor outcomes (Hou et al., 2021).

### Heart-type fatty acid binding protein (hFABP)

hFABP is a 15 kDa cytoplasmic protein that participates in fatty acid metabolism in the myocardium and neurons (Park et al., 2013). hFABP concentration in plasma was shown to be independently associated with stroke prognosis, as assessed 3 months after ischemic stroke (Park et al., 2013). Increased plasma levels of hFABP indicate acute cardiovascular dysfunction, which in turn is associated with neuronal injury (Park et al., 2013).

### D-dimer

D-dimer is a fibrin degradation product that gets released into the circulation by fibrinolysis of cross-linked fibrin; thus, it is used as a biomarker to monitor coagulation and fibrinolysis (Abd-Elhamid et al., 2016; Wang J. et al., 2020). During thrombosis, the plasma levels of D-dimer increase, which makes it a promising biomarker of hemostatic anomalies and thrombosis (Yao et al., 2019). D-dimer is measured in plasma or whole blood to estimate the infraction size, stroke progression, incidence of stroke, and clinical outcomes in ischemic stroke (Abd-Elhamid et al., 2016; Yao et al., 2019). Mixed evidence of the correlation between D-dimer concentrations and the prediction of poor prognosis makes it controversial to conclude with a high degree of confidence that D-dimer can be used as a prognosis biomarker (Yao et al., 2019; Zhang et al., 2019; Wang J. et al., 2020). However, several studies have shown a positive association between D-dimer levels and functional outcomes. For instance, high plasma levels of D-dimer from samples taken within the first 24 h after admission were correlated with unfavorable functional outcomes assessed using the mRS 1 month after admission (Abd-Elhamid et al., 2016). In another study, patients with ischemic stroke were followed up at 3, 6, and 12 months after discharge (Wang J. et al., 2020). A positive and dose-dependent relationship between the plasma levels of D-dimer and increased risk of poor functional outcome was observed. The positive correlation remained significant at 12-month follow-up, suggesting that D-dimer is a promising biomarker for long-term prognosis (Wang J. et al., 2020). Similar results were reported in another long-term study in which D-dimer levels measured by ELISA within 1 week of admission, predicted mortality after 1.2 years of follow-up (Feinberg et al., 1996). However, the predictive ability of D-dimer depends on blood samples taken on admission. Therefore, it is crucial to assess whether D-dimer measured at different time points after stroke could still predict a poor prognosis.

### Fibulin-5

Fibulin-5, found in the extracellular matrix and secreted by vascular cells, is a 66 kDa glycoprotein crucial for the assembly of elastic fibers and vasculogenesis (Hu et al., 2016; Elshony et al., 2021). Endothelial cells increase the expression of fibulin-5 in response to hypoxic conditions (Elshony et al., 2021). Fibulin-5 is considered a neuroprotective factor that improves neurological prognosis by increasing the blood-brain barrier permeability as a compensatory mechanism after ischemic stroke (Hu et al., 2016). Overexpression of fibulin-5 has been associated with improved clinical outcomes following reperfusion and has been regarded as a biomarker to predict severity and prognosis in both ischemic and hemorrhagic stroke (Hu et al., 2016; Elshony et al., 2021). Hypoxic stress, in ischemic stroke, and relative ischemia, in hemorrhagic stroke, induce fibulin-5 expression in endothelial cells *via* the hypoxia-inducible factor-1, which is an adaptive response to hypoxic conditions (Elshony et al., 2021). A positive correlation between fibulin-5 levels and functional outcomes measured using the mRS in a 3-month follow-up study in both ischemic and hemorrhagic stroke patients, suggested the ability of fibulin-5 to predict poor prognosis (Elshony et al., 2021). Similar results were reported in another 3-month follow-up study with ischemic stroke patients (Hu et al., 2016).

## Inflammation biomarkers

### Monocytic HLA-DR (mHLA-DR)

In ischemic stroke, the role of the inflammatory response has been previously established as the levels of several inflammatory biomarkers increased in stroke patients compared to their healthy counterparts (Aref et al., 2020). mHLA-DR is an inflammation biomarker used for injury-associated immunosuppression involved in the cross-talk regulatory mechanism between innate and adaptive immunity (Pfortmueller et al., 2017). Because mHLA-DR is associated with impaired monocyte function resulting in a low rate of antigen presentation, which leads to reduced lymphocyte response, it is considered a biomarker for immunosuppression induced by stroke (Mengel et al., 2019). In patients with ischemic stroke, low concentrations of mHLA-DA predicted poor functional outcomes measured using mRS after 3 months of symptom onset (Mengel et al., 2019).

### Interleukin-6 (IL-6)

IL-6 is an inflammatory cytokine that stimulates the release of prostaglandin E2 in the brain and is involved in the pathogenesis of several neurological disorders (Shaafi et al., 2014; Aref et al., 2020; Mosarrezaii et al., 2020). IL-6 acts as a secondary messenger in the communication between leukocytes, vascular endothelium, and parenchymal cells and participates in the response to neuronal damage (Mosarrezaii et al., 2020). Shortly after ischemic events, the levels of IL-6 significantly increase in the CSF and blood; this increase is an indication of early neurological deterioration and poor prognosis (Aref et al., 2020; Mosarrezaii et al., 2020). In a study with ischemic stroke patients, serum IL-6 levels were monitored at different time points (4, 8, 12, 24, 48, and 72 h, 7 and 12 days, 3 months, and 1 year) after stroke to assess the correlation between serum IL-6 with stroke volume, size of the infarction, and long-term functional outcomes (Waje-Andreassen et al., 2005). The study showed a positive relationship between IL-6 levels and these variables, demonstrating the promising use of IL-6 as a biomarker to assess long-term prognosis after ischemic stroke (Waje-Andreassen et al., 2005). Similar findings have been reported in more recent studies. For instance, poor functional outcomes (measured using the European stroke scale and BI) were associated with a high concentration of IL-6 after 3 months of hospital admission in samples taken within 36 h of the stroke event (Mengel et al., 2019). A positive relationship between stroke severity (NIHSS), physical disability (mRS), and IL-6 serum levels after 5 days, 3 months, and 1 year was reported (Shaafi et al., 2014). Furthermore, elevated levels of IL-6 in both CSF and blood have been associated with an increased risk of fever, which in turn causes tissue damage after ischemic stroke (Aref et al., 2020). Poor functional outcomes (NIHSS and mRS) after a 3-month follow-up of ischemic stroke patients were associated with high concentrations of IL-6 in serum measured in samples taken within the first 24 h of symptom onset (Aref et al., 2020).

### C-reactive protein (CRP)

The acute-phase CRP, also known as high-sensitive CRP, is a non-specific biomarker of inflammation rapidly produced in the liver in response to tissue injury or infection (Yu et al., 2017). Cell death, brain injury, and blood-brain barrier disruption have been associated with elevated concentrations of CRP post-stroke (Gu et al., 2022). High levels of CRP have been reported in the first 48 h after symptom onset, and they remained elevated on day seven and even after three to 6 months post-stroke (Mengozzi et al., 2020). Poor functional outcomes assessed with the mRS and stroke recurrence were predicted by high levels of CRP in patients with ischemic stroke in a 3-month follow-up study (Gu et al., 2022).

### Mannose-binding lectin (MBL)

The complement MBL is an important element of the innate immune response (Osthoff et al., 2011). MBL activates the complement system through MBL-associated serine proteases to initiate complement activation *via* the MBL pathway (Huang et al., 2016). Complement activation is known to have detrimental effects on the pathogenesis of ischemia/reperfusion injury through the production of free radicals, stimulation of the coagulation cascade, and tissue damage (Osthoff et al., 2011). Complement activation may also result in the stimulation of potent inflammatory reactions that lead to tissue damage (Zhang et al., 2015). MBL is an important role player in brain injury as this molecule regulates and coordinates several pathogenic pathways involved in ischemic brain injury (Neglia et al., 2020). In patients with acute ischemic stroke, low levels of MBL in serum were associated with smaller infarct volume and improved functional prognosis (measured by mRS) in a 3-month follow-up study (Osthoff et al., 2011). Furthermore, higher serum MBL levels in patients with ischemic stroke predicted poor outcomes in the mRS in another study (Zhang et al., 2015).

### 25-hydroxyvitamin D (25-(OH)D)

25-(OH)D is a well-known biomarker to assess the status of vitamin D. Reports indicate that vitamin D deficiency can induce brain damage and cause functional impairment. Macrophage and lymphocyte activity in atherosclerotic plaques is influenced by low vitamin D levels, and this phenomenon promotes inflammation in artery walls (Tu et al., 2014). As vitamin D has anti-inflammatory activity, high levels of vitamin D have been shown to promote recovery after stroke. For instance, a study showed a negative correlation between vitamin D and unfavorable outcomes at discharge (mean hospital stay of 31 days) assessed using the NIHSS (Wang Y. et al., 2014). Another study assessing the level of disability using the mRS after 3 months of the stroke event found that a poor outcome measured by the mRS was associated with low serum levels of 25-(OH)D (Tu et al., 2014).

### YKL-40

YKL-40, also known as chitinase 3-like-1 and human chitinase-like protein, is an inflammatory biomarker secreted by monocytes that infiltrate vessel walls upon brain injury (Park et al., 2012; Nadeem et al., 2019). YKL-40 activates modulators of cell proliferation, survival, migration, and adhesion such as mitogen-activated protein kinase (MAPK) and phosphoinositide 3-kinase (PI3K), which increases the chances of a vascular event (Li et al., 2022). In patients with ischemic stroke, a positive correlation between YKL-40 levels and stroke severity (NIHSS) and a poor functional outcome (mRS) was observed in a 3-month follow-up study (Park et al., 2012). YKL-40 long-term prognosis was evaluated after 3 months and one to 5 years using the mRS in patients with ischemic stroke or transient ischemic attack, and an independent association between YKL-40 levels with stroke recurrence, and an unfavorable outcome in the mRS were reported (Li et al., 2022).

## Oxidative stress biomarkers

### Superoxide dismutase (SOD)

SOD is an oxidoreductase that catalyzes dismutation of superoxide radicals into O2 and H2O2 (Fukai and Ushio-Fukai, 2011). Molecular and biochemical studies have characterized three isoforms of superoxide dismutase, namely SOD1 (Cu/ZnSOD) in the cytoplasm, SOD2 (MnSOD) in the mitochondria, and SOD3 (ecSOD) in the extracellular matrix and on the cell surface. SOD1 and SOD2 are present in practically all cells, while SOD3 is found in selected tissues, including blood vessels, lungs, kidneys, and hearts, as well as extracellular fluids including blood (Fukai and Ushio-Fukai, 2011). After ischemic stroke, the SOD activity of the brain decreased dramatically and gradually decreased with infarct size (Spranger et al., 1997). Low serum levels of SOD were found within 24 h of stroke onset and the level of functional impairment, measured by the Scandinavian Stroke Scale (SSS), and the size of the infarction, were negatively correlated with SOD activity.

### Malondialdehyde (MDA)

MDA is a lipid peroxide produced when polyunsaturated fatty acids are peroxidized (Barrera et al., 2018), and it is markedly elevated during the acute phase and persistently maintained during the late phase of stroke (Nakane et al., 2015). When subjects were compared in the acute, subacute, and chronic phases of stroke recovery, a study found that the plasma lipid peroxide level significantly increased over time (Alexandrova et al., 2003). Serum MDA levels of post-stroke patients were significantly higher than their healthy counterparts (Bir et al., 2006; Corrêa Mde et al., 2008). The primary source of MDA production in post-stroke patients is still unclear. Increased lipid peroxide and MDA levels, on the other hand, could be caused by spontaneous reactive oxygen species (ROS) generation by peripheral phagocytes (Alexandrova et al., 2003), extracellular hydrogen peroxide production by paretic muscles during denervation (Jackson and McArdle, 2016), or associated comorbidities such as diabetes, hypertension, and hyperlipidemia (Cheng et al., 2017). MDA, measured in serum, has been proposed as an oxidative stress marker (Ohkawa et al., 1979). In the follow-up evaluation of stroke patients, the assessment of the short-term outcome and disability was measured 3 months after stroke using the mRS (Elsayed et al., 2020). Serum samples were taken within 24 h after admission (Elsayed et al., 2020). An elevated serum MDA level at admission was independently associated with a poor functional outcome, as assessed with the mRS, following an ischemic stroke at a 3-month follow-up (Elsayed et al., 2020).

## Metabolic biomarkers

### Retinol binding protein 4 (RBP4)

Among the cytokines produced primarily by adipose tissues, RBP4 is the only protein specifically responsible for delivering retinol (vitamin A) to tissues and blood (Quadro et al., 1999). In the brain, retinol is oxidized to retinoic acid, which has been shown to contribute to neurogenesis and neuroplasticity during both the early stages of growth of the central nervous system and adult functions of the brain (Plane et al., 2008; Shearer et al., 2012). The anti-apoptotic properties of retinoic acid have also been demonstrated to reduce cerebral ischemia by regulating inflammation (Harvey et al., 2004; Choi et al., 2009). RBP4 levels have been examined in the context of ischemic stroke (Sasaki et al., 2010). Patients with cerebral infarction had higher levels of RBP4 compared to healthy controls (Llombart et al., 2016). Elevated levels of RBP4 have been found among women who had a stroke compared to those who did not have a stroke during the discovery phase based on data from the Women’s Health Initiative (Rist et al., 2018). Moreover, a study involving a 3-month evaluation where blood samples were collected within 48 h after the stroke, found that serum RBP4 levels were significantly higher in patients with acute stroke than in those in the healthy controls, and were correlated with stroke severity and poor neurological outcomes as measured using the NIHSS (Liu and Che, 2019).

### Glycated hemoglobin (HbA1c)

HbA1c has been recognized as abnormal hemoglobin in diabetes (Rahbar et al., 1969). The discovery of HbA1c led to numerous small studies correlating glucose measurements to HbA1c, resulting in the idea that it could be used objectively to measure glycemic control (Rahbar et al., 1969). Glycated hemoglobin (HbA1c) is a useful biochemical indicator for measuring glycemic control over the past 3 months because the values are not affected by short-term transient fluctuations in blood glucose levels (Bao and Gu, 2021). A positive association was found between blood glucose levels and the levels of HbA1c at the time of admission for stroke (Toni et al., 1994; Mohanty et al., 2000; Kagansky et al., 2001). The triggering of inflammatory pathways may play a role in determining the size or severity of infarcts caused by uncontrolled blood glucose (Toni et al., 1994; Mohanty et al., 2000; Kagansky et al., 2001). Each unit increase in HbA1c (continuous) and HbA1c ≥ 6.5% were found to be associated with an increased risk of poor functional outcome defined by the mRS (Wang H. et al., 2019) after ischemic stroke. Thus, these findings suggest that HbA1c may be a useful biomarker for monitoring or predicting poststroke prognosis, allowing appropriate management to be instituted (Bao and Gu, 2021).

## Tissue remodeling and apoptosis biomarkers

### Matrix metalloproteinase-9 (MMP-9)

Matrix metalloproteinases (MMPs), a class of proteolytic zinc-dependent enzymes, have been associated with a variety of pathophysiological processes including systemic inflammation, atherosclerosis, and central nervous system diseases (Creemers et al., 2001; Galis et al., 2002). MMP-9 (gelatinase B, 92-kDa collagenase) is a well-studied enzyme in ischemic stroke, and its expression is rapidly upregulated after cerebral ischemia (Romanic et al., 1998; De Marchis et al., 2013). Several clinical studies have found a correlation between circulating MMP-9 levels, disease severity, and infarct volume in the hyperacute phase (Rosell et al., 2005; Demir et al., 2012) and late hemorrhagic infarction between days five and seven after stroke onset (Montaner et al., 2001). A higher serum MMP-9 level in ischemic stroke was associated with an increased risk of physical disability (mRS) in a 3-month follow-up study (Zhong et al., 2017). Therefore, MMP-9 may be a potential prognostic biomarker for ischemic stroke (Ramos-Fernandez et al., 2011).

## Infection biomarker

### Lipopolysaccharide-binding protein (LBP)

LBP is an acute-phase protein that is critical for endotoxin signaling (Schumann and Zweigner, 1999; Bas et al., 2004). In endotoxin response, LBP is synthesized by gastrointestinal and liver epithelial cells (Schumann and Zweigner, 1999). As a part of the innate immune response, LBP plays an important role in both Gram-negative and Gram-positive infections (Zweigner et al., 2006). In addition to binding directly to endotoxins, LBP facilitates their transport to CD14 receptors, a receptor that detects patterns, and stimulates the production of proinflammatory cytokines by binding to toll-like receptors promoting the binding of lipopolysaccharides to these receptors. It has been observed that the levels of LBP and sCD14 in blood increase during an ischemic stroke (Klimiec et al., 2016; Hoffmann et al., 2017). In a study measuring plasma LBP levels on days one to four after stroke, it was found that the highest level of plasma LBP after stroke was observed on day four in patients with stroke-associated pneumonia (Hoffmann et al., 2017). A high level of LBP and sCD14 was associated with poor functional prognosis (Mengel et al., 2019). A moderate correlation was found between plasma LBP and acute lesion volume in stroke patients without infections (Hotter et al., 2019). Levels of LBP in stroke patients with infection are increased (Wartenberg et al., 2011). In another study, LBP was predictive of poor prognosis 3 months after stroke, with high levels of LBP associating with poorer outcomes in the mRS (Mengel et al., 2019).

## Discussion

In this review, we have summarized and discussed several prognostic biomarkers, collected from laboratory and clinical neuroimaging/neurophysiology studies, for predicting functional recovery after stroke. Neuroimaging/neurophysiological studies reveal the importance of structural integrity (measured by MRI) and functional integrity (measured by MEP) of the CST in the prediction of poststroke motor recovery. The damage to structural motor connections and functional disturbances after stroke, in the form of the redistribution of cortical rhythmic powers, pathological alteration of cortical evoked potentials and change of intra-/interhemispheric coherent activities among the motor system, are also indicative of recovery potentials. In addition, several molecular biomarkers have been implicated in predicting functional recovery using blood samples taken days, months, and even a year after stroke onset. The molecular biomarkers that stand out as potential candidates for monitoring stroke prognosis include CRP, fibrinogen, IL-6 for the measurement of inflammation, NfL for neuroaxonal damage, and SOD activity for oxidative stress. All prognostic biomarkers described in this review are relevant to neural structure (at the molecular, cellular, and systems levels) and functions (in terms of neuronal functional response to injury, excitability, activation levels, and connectivity) poststroke. The evidence from systems neuroscience studies largely supports that the initial structural injury to the motor system and the level of interhemispheric imbalance, as reflected by various functional measures, are predictive of functional outcomes, whereas the evidence from molecular/cellular neuroscience research supports that the initial damage to neuronal and glial cells as well as associated functional reactions to pathological injuries, such as inflammation and oxidative stress reactions, are relevant with functional prognosis (Figure 1).

Most studies included in the review assessed functional recovery after ischemic stroke and very few studies targeted hemorrhagic stroke. Furthermore, the majority of the studies discussed in this review used blood samples taken at baseline (typically within 24–48 h after symptom onset) and considered the amount of functional recovery at more than 3 months after symptom onset as the outcome (Montellano et al., 2021). The analysis of the biomarkers at the time of functional recovery assessment is missing. Many studies were cross-sectional in nature and the association relationship did not represent a longitudinal change across the time since onset of stroke. Even though some neuroimaging and neurophysiological biomarkers were repeatedly measured, a longitudinal association between the change of biomarkers and functional recovery was not clearly reported or consistently found in previous studies. Repeated measures of biomarkers are essential to reveal the mechanism underlying recovery. For example, a higher level of motor-related brain activations in fMRI at the acute stage (within 1 week after stroke) was associated with a favorable outcome at the chronic stage, and the association was not significant when using the fMRI results measured at 2 weeks after stroke (Rehme et al., 2015), indicating the importance of measurement time points in biomarkers research and that the time points after onset of stroke should be clearly defined. In addition, the longitudinal correlation between the change of biomarkers and functional recovery across time may not be consistent with the correlation between baseline biomarkers values and the amount of functional recovery. For example, a study by Ward et al. (2003), demonstrated that a longitudinal association between the change of brain activation levels and the amount of functional recovery was in a negative direction, which was opposite to the results of a study by Rehme et al. (2015), using baseline activation levels only. These findings highlighted the unique prognostic features of biomarkers collected at different phases after stroke, because different recovery mechanisms, either the restitution of interhemispheric balance or the evolution of the compensatory mechanism of the contralesional hemisphere, may underlie the recovery of individual stroke patients. Thus, to monitor poststroke prognostication, it is pivotal to identify and validate biomarkers that could not only accurately predict functional prognosis with a single baseline value, but also correlated with progressive recovery poststroke in a longitudinal manner. Robust biomarkers for the prognosis of poststroke recovery are therefore needed, which are helpful not only for clinicians to predict the individual recovery trajectory poststroke, but also contribute to further development of conceptual models of poststroke prognostic recovery.

Although a variety of mixed biomarkers has been validated, the predictive potential of biomarkers is typically based on correlation and regression analyses, without out-of-sample evaluation and validation. In addition, a combined approach using multimodal biomarkers for prognostic prediction has rarely been studied in previous literature. The PREP algorithm is an example of combining neuroimaging, neurophysiology, as well as clinical measures for motor recovery prediction. However, no laboratory-based molecular biomarker was included when developing this algorithm (Stinear et al., 2012). The use of a single biomarker for prognosis is not enough for the accurate prediction of functional outcomes. Instead, a combination of multimodal biomarkers would increase prediction capability.

New and more accurate biomarkers for prognostic functional recovery poststroke are needed. Toward this end, a potential approach is to use a multivariate model, whereby both molecular biomarkers and neuroimaging/neurophysiological indexes that are relevant to the structure and functions of the neural system should be adopted. This approach may potentially provide a more holistic understanding of the poststroke functional recovery process. On one hand, neuroimaging and neurophysiological studies contribute to the initial topographic mapping of the structural damage and functional alterations induced by the stroke event to predict recovery, while the general-systemic indexes collected *via* molecular and cellular neuroscience approaches evaluate the level of the initial damage to neurons and the pathophysiological response to the injury. On the other, longitudinal measurements of neuroimaging, neurophysiological and general-systemic indexes could support functional recovery monitoring over time. Studying the longitudinal regression between neuroimaging/neurophysiological measurements and molecular biomarkers over time could contribute to the prediction of functional recovery to assist clinical practitioners and researchers in identifying distinct recovery patterns of individual stroke patients and offering optimal rehabilitation choices to facilitate their neural recovery.

## Limitations

The review is not free from limitations. On one hand, our review only focused on prognostic biomarkers that were predictive of functional outcomes based on the evaluation of overall neurological deficits (NIHSS), physical disability, and life dependence (mRS and BI), or motor domain (FMA and ARAT). We have excluded biomarkers associated with cognition and language-specific outcomes as well as clinical endpoints such as mortality and reoccurrence of stroke. On the other hand, our review descriptively summarized and discussed the literature without a quantitative data synthesis, due to great heterogeneity in the study design, analysis methods, and statistical protocols used across studies.

## Conclusion

Molecular and systems neuroscience approaches have provided several promising biomarkers with prognostic values for the prediction of functional recovery after stroke. Future studies are recommended to incorporate a combination of multiple biomarkers from big data and develop supervised machine learning algorithms using data mining methods to predict the recovery potential of patients after stroke in a more precise way.
